# AI-supported clinical decision-making: in silico simulation of physician-AI interactions

**DOI:** 10.3389/fdgth.2025.1697825

**Published:** 2026-01-09

**Authors:** Amun Hofmann

**Affiliations:** 1 FIFOS—Forum for Integrative Research & Systems Biology, Vienna, Austria; 2 Department of Vascular and Endovascular Surgery, Klinik Ottakring, Vienna, Austria

**Keywords:** artificial intelligence, decision support systems, decision-making, human-AI interaction, simulation

## Abstract

**Objective:**

While the integration of modern AI systems in clinical practice is currently in the process of transforming how medicine is being practiced, the focus of most research activities lies on AI-associated efficacy and safety. However, the interplay between human agents and AI systems will equally shape the actual impact of such systems.

**Methods:**

This study simulated human decision-making using 27 agents characterized by varying levels of competence, certainty, and trust. Agents completed binary and three-option decision tasks, both with and without AI assistance. AI models varied in competence (0.3–0.9) and, in some simulations, included confidence signals to influence human trust dynamically. Each scenario involved 10,000 simulated decisions per agent. In AI-assisted conditions, decisions were modulated by the agent's baseline trust and, in the conditional trust setting, the AI's expressed confidence.

**Results:**

AI support significantly improved decision accuracy for most agents, especially those with high competence but low certainty. In binary tasks, agents showed up to 150% relative improvement in decision accuracy with AI competence ≥0.6. In three-option tasks, even lower-performing AI (e.g., 0.4 competence) enhanced decision results. Conditional trust simulations showed further gains, particularly among agents with moderate baseline trust, as dynamic trust adjustments based on AI confidence reduced over-reliance on poor AI recommendations.

**Discussion:**

Results demonstrate that AI assistance, particularly when paired with confidence calibration, enhances human decision-making, especially for uncertain or moderately skilled users. However, over-trusting low-competence AI can impair outcomes for high-performing agents. Tailored AI-human collaboration strategies are essential for optimizing clinical decision support.

## Introduction

Artificial intelligence (AI) is arguably revolutionizing clinical decision-making. A recent review highlights AI's role in facilitating diagnosis and prognosis, for example supporting surgical interventions for conditions such as lung cancer through deep learning models and optimizing intraoperative decisions via patient triage in emergency settings (1). The integration of modern AI systems and robotics is further gaining traction, potentially improving intraoperative assessments and decision (2). AI has been even investigated regarding its usefulness in the context of shared (patient-physician) decision-making (3). However, regulatory gaps and limited user perspective data pose adoption barriers (1).

Nevertheless, opportunities, performance, and challenges of modern AI systems and tools are one aspect within the broader discourse, while its acceptance by its (potential) users remains a focal but less investigated element. Research on the acceptance of emerging technologies has been ongoing for several decades. The number of studies on AI acceptance recently increased, with (historic) theoretical models such as the Technology Acceptance Model (4) commonly being used to predict behavioural intentions through factors such as perceived usefulness, trust, and effort expectancy, though newer models such as AI Device Use Acceptance Model (5) potentially offer more detailed insights by accounting for both willingness and rejection (6). Yet, much of the research lacks external validity due to limited evaluation of actual behaviour and a shortage of naturalistic studies, highlighting the need for more comprehensive, real-world investigations into AI uptake across diverse contexts (6).

Domains other than clinical decision-making have been previously under the scope regarding the impact of AI acceptance. In public policy, for example, Robles & Mallinson argue that trust in AI governance might have been previously overlooked and that it should be considered a foundational principle alongside transparency and risk mitigation. Drawing on public opinion data from a nationally representative U.S. sample, the authors reveal widespread support for AI development, but also significant concerns about privacy, bias, and civil rights concerns that vary across demographic and political lines (7). Research on consumer acceptance of AI advice in high-stakes situations revealed a consistent preference for human over AI interactions, not because AI is seen as inferior, but due to a deeper human tendency. The studies show that this preference intensifies in high-stakes contexts like healthcare and is further influenced by self-threats, which help explain resistance to AI advice beyond general negative perceptions (8).

While research on the effectiveness and safety of AI systems in clinical decision-making remains highly relevant and will certainly reshape the current landscape, understanding human-AI interactions within this context will be just as important. The present research investigates this in the course of subsequent simulation experiments.

## Methods

### Agent description

In summary, 27 synthetic agents were examined regarding human-AI complementarity, each defined by a unique profile of three traits: competence (o_a_), certainty (c_a_), and baseline trust in AI (t_a_). Each trait took one of three values {0.3, 0.6, 0.9}, yielding 3 × 3 × 3 = 27 distinct agent types. AI in this investigation refers to a AI prediction model that is assumed to be validated for the respective use case. Competence o_a_ represents the probability that the agent's initial (primary) decision d_i_^(1)^ matches the true label y_i_. Certainty c_a_ is the probability that the agent does not reverse their primary decision due to self-doubt. Baseline trust t_a_ reflects the agent's *a priori* willingness to follow an AI recommendation when it conflicts with their own judgment.

### Simulation studies

The simulation studies follow a multi-step workflow. First, a binary decision task was simulated with a 50–50 distribution of true labels with a primary decision and secondary decision. This was conducted without an assisting AI prediction model and subsequently with AI models that had accuracies ranging from 0.3 (30% correct decisions) to 0.9 (90% correct decisions) in 0.1 increments. Subsequently a decision problem with 3 potential options with even distribution of true labels was simulated, again with the same AI systems. All decision problems included 10,000 decisions per agent. The simulated decisions were based on entirely theoretical/abstract binary or ternary choice paradigms, with true labels distributed evenly. No real clinical cases, vignettes, or examination question banks were used; instead, decisions were generated probabilistically to isolate the effects of agent traits and AI assistance in a controlled environment. This approach allows for high-volume iterations but may limit direct translatability to specific clinical scenarios.

### Agent-AI interaction

In the first instance (no AI support), decisions were determined by the fixed human competence level (i.e., the probability of selecting the correct answer) and the fixed confidence parameter; when confidence was low, individuals were more likely to revise their initial decisions, simulating cognitive doubt or inconsistency. In the second scenario (human-AI collaboration), both the human and an AI agent independently generated decisions based on their respective competence levels. When discrepancies arose between the two, a trust parameter governed the probability that the human would adopt the AI's recommendation. Supplementary Figure S1 illustrates the process for the binary problem.

### Conditional trust

The conditional trust simulation was based on a binary decision-making process under two distinct conditions: decisions made solely by a human agent as in the previous simulations and decisions made with AI assistance. In the conditional-trust condition, the AI provided a prediction p_i_ (correct with probability *α* ∈ {0.3, 0.4, 0.5, 0.6, 0.7, 0.8, 0.9}) together with a confidence score *κ*_i_ drawn from N(0.8, 0.1) when correct and N(0.3, 0.1) when incorrect (clipped to 0, 1). Effective trust on trial i was calculated as $\tau_{1}\;=\;1\;/\left(1\;+\;exp[-10\;\times \;(t_{a}\;\times \;\kappa_{1}\;-\;0.4)]\right)\;$ where t_a_ ∈ {0.3, 0.6, 0.9} is the agent's baseline trust. When p_i_ differed from the human's post-uncertainty decision d_i_^(2)^, the agent adopted the AI's recommendation with probability *τ*_i_ (i.e., a Bernoulli draw was performed for each conflicting trial). This multiplicative-then-sigmoid transformation ensures that high-confidence correct AI advice yields substantially higher effective trust than low-confidence or incorrect advice, while preserving meaningful differences across the three discrete baseline-trust levels. The conditional trust assumes well-calibrated AI confidence instead of real-world systems that may exhibit miscalibration, potentially altering trust dynamics.

### Analysis

All simulations and visualizations were conducted using Python v3.12.11. Descriptive analyses were performed to compare agents and experimental set-ups.

## Results

### Binary decision problem

Simulating a decision problem with 2 answers, one correct and one wrong, with a primary and secondary decision process without AI assistance, resulted in varying performance between the different agents. Intuitively, agents with high competence and high certainty in their decisions outperform other agents with correct decisions in over 80% of cases. The worst performance was observed with agents that have a combination of either high competence and low certainty or low competence and high certainty as both come up with correct decisions in around 33% of cases.

**Figure 1:**
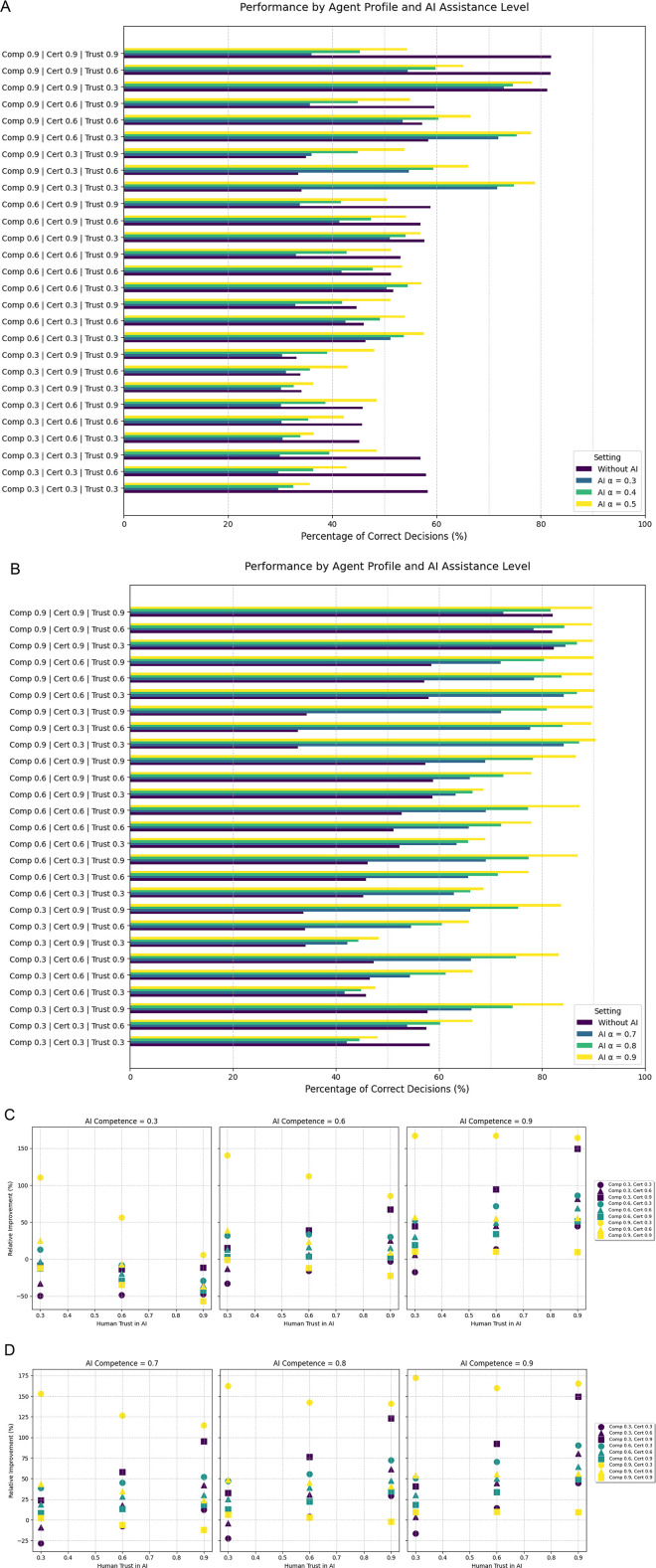
Illustrates the results from the first simulation experiment in a binary decision task. “AI *α*” in the legend in **(A,B)** refers to the AI system's competence. **(C,D)** Depict the relative improvement for each agent depending in the AI system's competence. (Comp: human competence, Cert: human certainty, Trust: human baseline trust in the AI system).

With the introduction of AI models that support the secondary decision process, performances changed for all agents. When the AI prediction model featured a competence level of at least 0.6, its assistance generally led to an increase in the number and percentage of correct decisions across various agent types. The biggest increases can be found in agents with high competence but low certainty, where, depending on the AI competence, correct decisions can increase over 150%. The extent of this improvement is influenced by both the human agent's characteristics (competence and trust in AI) and the AI's own competence level. Higher AI competence consistently resulted in greater relative improvement in decision accuracy, comparing the same agents across different AI competence levels. Conversely, highly competent and certain human agents experienced less improvements, and in some cases, particularly with lower AI competence, their performance decreased with high trust in the AI (Figures 1A,C).

While results and improvements of AI assisted decision-making are to a certain degree agent specific, incorporating an AI system with a moderate competence (0.6) improved the results of a large majority of agents. Across all degrees of trust in the prediction model, 22 of the 27 agents (81.5%) improved their predictions in each instance. With increasing AI competence (0.7–0.9), 22–26 agents could improve their performance by incorporating the AI prediction model (Figures 1B,D)

### Decision tasks with three options

**Figure 2:**
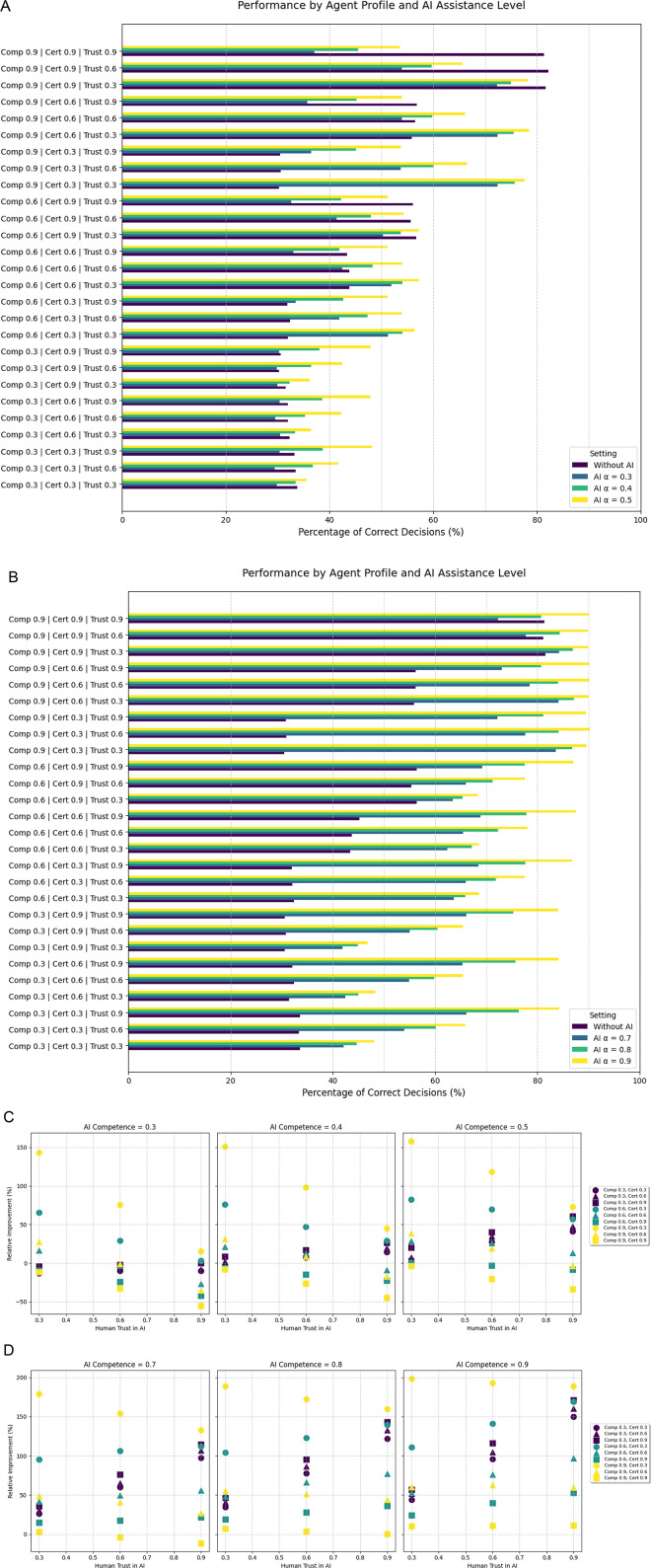
Illustrates the results from the second experiment in a tertiary decision problem. “AI *α*” in the legend in **(A,B)** refers to the AI system's competence. **(C,D)** Depict the relative improvement for each agent depending in the AI system's competence. (Comp: human competence, Cert: human certainty, Trust: human baseline trust in the AI system).

In a decision problem with 3 available answers, one right and two wrong, overall performance of all agents decreased, even though agents with high competence and certainty retained most of their performance compared to the binary decision task. Compared to a binary decision task, most agents had the correct decision in 30%–35% of cases, except for combination with moderate to high competence and moderate to high certainty (Figures 2A,B).

In a decision problem with three potential answers, the effects of AI support diverged from the binary setting. Improvements of approximately 200% were reached (high competence—low certainty agent). At an AI competence level of 0.5, 20 out of 27 agents (74.0%) improved their decisions, while at 0.7–0.9 between 25 and 27 agents improved their decisions. In general, in decision problems with 3 options even AI models with lower competence improved the decisions across all agents. For example, support with an AI model that had a competence of 0.4 had similar effects as the prediction model with a competence of 0.6 in the binary decision task (Figures 2C,D, Table 1).

**Table 1:** Depicts median (Q1, Q3) of relative improvements in decision making across the 27 distinct agents synthesized in the simulation studies.

AI competence	Two options	Three options	Conditional trust
0.3	−18.9% (−36.1, −7.2%)	−5.3% (−12.2, 12.0%)	17.1% (−1.2, 32.9%)
0.4	10.1% (−3.4, 27.9%)	9.8% (−3.7, 30.5%)	19.5% (4.0, 45.2%)
0.5	28.1% (3.7, 52.5%)	27.3% (6.0, 55.3%)	28.5% (7.4, 51.7%)
0.6	15.5% (0.3, 31.2%)	37.0% (16.0, 82.0%)	38.4% (9.5, 55.9%)
0.7	26.1% (10.6, 45.4%)	51.4% (28.5, 102.8%)	39.7% (11.6, 64.1%)
0.8	40.0% (17.6, 61.8%)	61.8% (41.7, 124.2%)	49.7% (14.3, 76.9%)
0.9	50.4% (25.7, 76.9%)	78.5% (55.1, 147.0%)	58.2% (21.6, 88.4%)

### Conditional trust setting

**Figure 3:**
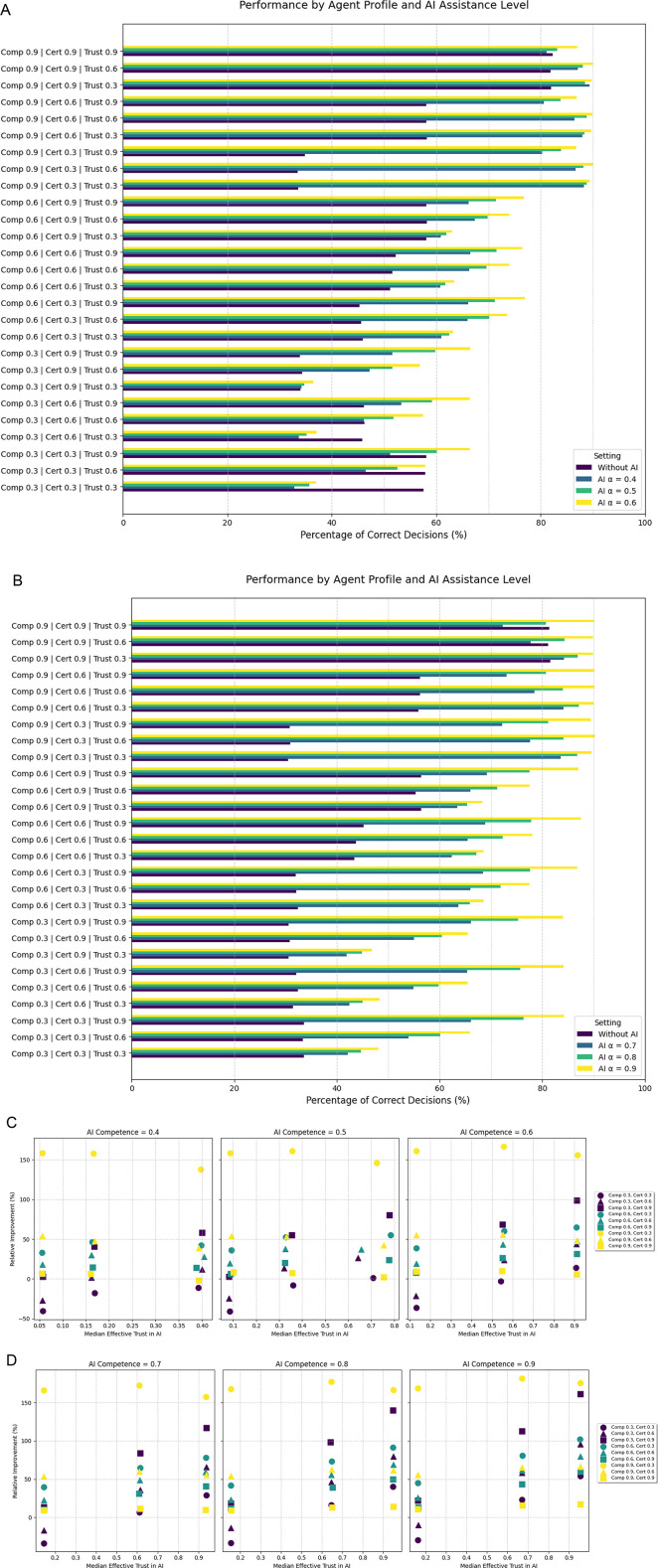
Illustrates the results from the third experiment in a conditional trust setting. “AI *α*” in the legend in **(A,B)** refers to the AI system's competence. **(C,D)** Depict the relative improvement for each agent depending in the AI system's competence. (Comp: human competence, Cert: human certainty, Trust: human baseline trust in the AI system).

In a further simulation the effects of conditional trust were investigated. Here, the trust the human agent placed in the AI prediction was additionally shaped by the confidence of the AI system in its own prediction, where the AI prediction model would indicate strong confidence in true predictions and weak confidence in wrong predictions. Compared to the initial binary decision task, the integration of an AI system generally improved the performance across all agents, even with AI system that operate with low to moderate competence and median improvements were higher in all instances (Figures 3A,B).

Apart from the decisions, the effective trust of the human agent in the AI system in this setting is shaped by both the baseline human trust in AI as well as the AI's reported confidence in it's prediction. Figures 3C,D illustrate the median effective trust of the agents for different AI competence levels. Human agents with low trust in the AI system on average have even lower trust when a AI confidence is provided. On the contrary, agents with moderate to high baseline trust increase their willingness to consider AI predictions when a predictive uncertainty is provided. This subsequently improves final predictions. For example, whereas two agents with moderate trust in AI in the initial binary decision task had a decline in results after implementing an AI system with a competence of 0.6, there is only one agents in the same instance when AI confidence is provided. With increasing AI competence, results further improve as median effective trust increases. Table 1 summarizes the results of all simulations stratified by AI competence level.

## Discussion

This study simulates human decision-making in binary and three-option tasks, evaluating how AI assistance influences performance. In the binary task (one correct, one incorrect answer), agents with both high competence and high certainty achieved over 80% correct decisions, while those with mismatched competence and certainty performed poorly (∼33%). Introducing AI to support decisions improved accuracy, particularly for high-competence, low-certainty agents, with performance increases up to 150%. The benefit of AI support was closely tied to both the agent's trust in AI and the AI model's competence, with diminishing or even negative effects for highly competent agents when AI competence was low. In three-option tasks, overall accuracy declined, but AI support still led to notable improvements. Even lower-competence AI models (e.g., 0.4) improved outcomes similarly to higher-competence models in binary tasks. At AI competence levels of 0.7–0.9, nearly all agents improved. A further simulation introduced conditional trust, where human reliance on AI also considered the AI's confidence in its prediction. This mechanism improved decision outcomes across all agent types, especially for those with high and moderate trust, by boosting effective trust when AI confidence was high. Overall, integrating AI, especially with predictive confidence, consistently enhanced human decision-making.

It should be noted that the attributes of the human agents and AI systems are not necessarily picked to exactly replicate real-world situations but provide an opportunity to investigate the potential interplay of different factors and varying degrees in a controlled environment. The abstract nature of the decision substrate may amplify observed human-AI complementarity in simpler tasks, whereas real clinical substrates (e.g., evolving patient data) could introduce additional variability, as seen in sequential decision chains. Especially in clinical medicine it will also be rare to see both agents that continuously reach correct decisions in 30% or 90% of cases, depending on the given task. Furthermore, the correct answer in the in silico experiments of this investigation should be less interpreted as a definitive truth, but rather as the choice with the highest likelihood of leading to a desired outcome. The results might also be partially transferable to other domains regarding human-AI interactions and are not exclusive to clinical medicine. However, decision problems with two (drug A vs. drug B) or three options (open surgery, minimal invasive technique, or conservative treatment) are rather common in clinical medicine and therefore offer a relevant baseline for further investigations. Similarly, the conditional trust simulation touches the still growing integration of explainable AI (XAI) in medicine, where explanations for given predictions might further shape human trust in the underlying system (9, 10). Bauer & Michalowski recently argued in a review about XAI system evaluations in a medical context that the interaction between clinician and AI requires more attention in future research (11). The hereby presented research aims at doing so.

The simulation results provide insight into how AI assistance interacts with human decision-making traits (competence, certainty, and trust in AI systems) across different task complexities. In binary decision problems, highly competent and certain agents consistently performed best even without AI support, based on their internal capabilities. However, agents with either low certainty or mismatched competence and certainty demonstrated significant room for improvement. This fits well with a recently published study where high self confidence, apart from formal education, was the most relevant predictor for diagnostics performance in clinicians (12). When AI models were introduced, particularly those with moderate to high competence (≥0.6), these agents experienced the largest relative gains. This suggests that AI systems can be especially valuable in offsetting human cognitive limitations such as doubt or inconsistency. Interestingly, agents with high competence and high certainty benefited less from AI support and, in cases of excessive trust in low-performing AI, sometimes experienced performance declines. This finding underscores a key limitation of AI-human collaboration: over-reliance on AI can undermine good human judgment when the AI system is suboptimal. Thus, trust must be calibrated not only to the AI's presence but also to its reliability.

In three-option decision problems, overall accuracy declined, but the benefits of AI assistance persisted even with lower-competence models. This may reflect the increased cognitive load of multiclass decisions, where human agents can benefit more from external guidance. The conditional trust model further emphasized the value of explainability in AI: when AI confidence was disclosed, effective human trust adapted accordingly. Agents with initially moderate trust increased their reliance on AI when it showed high confidence, improving outcomes. Conversely, those with low baseline trust became more sceptical when AI confidence was low, leading to better avoidance of incorrect guidance. Overall, the results highlight the potential of adaptive AI support, particularly when AI systems communicate predictive confidence, to enhance decision quality across diverse user profiles and task complexities. Effective collaboration hinges on aligning AI capability with human traits and trust dynamics.

The simultaneous introduction of recent AI advances in several domains of clinical medicine brought many challenges for its potential users (physicians). This ranges from ethical consideration (13), to practical tasks such as learning how to interact with large-language models (14). Bakken adequately summarized in a recent editorial that clinical decision-making remains a central goal in biomedical and health informatics, with AI advancements continuing to support efforts to improve healthcare quality and equity (15). The integration of modern AI systems into clinical decision support systems offers transformative potential for enhancing patient care, clinical decision-making, and innovation through technologies like machine learning, natural language processing, and deep learning (16). However, Steyvers & Kumar formulated three key challenges for AI-assisted decision-making. These include understanding when and how AI should complement human input, accurately assessing human mental models of AI, and optimizing design choices in human-AI interaction to prevent overload or misuse (17).

This study addresses all three at least partially by exploring complementarity by analysing when AI assistance improves decision outcomes across varying human agent attributes, examining mental models through differing levels of agent trust in AI, and informing interaction design by testing how decision context (binary vs. tertiary) impacts reliance on AI. These simulations provide a controlled framework for understanding and optimizing human-AI decision-making. And as with most interventions, one-size fits all approaches might not improve outcomes for every individual. While individualized treatment strategies and personalized medicine are becoming the norm, partially fuelled by modern AI systems, it might be important to understand and explore what agents benefit from integrating AI systems in their clinical decision-making, specifically in what environment. As shown, predictive abilities are only one of multiple factors that determine outcome in an AI-supported setting. There might be situations where, for example, a less experienced, uncertain physician might benefit from a collaborative decision process whereas it could be detrimental for an experienced physician in the same situation. This is supported by recent empirical research showing that clinician acceptance of AI varies with experience and perceived risk. Senior clinicians were more accepting when perceived risk was low, while junior clinicians showed higher acceptance when they viewed AI as high-risk. These findings highlight how both experience and risk perception interact to shape AI adoption in clinical practice, aligning with our simulation's emphasis on tailoring AI support to individual traits (18).

There is an emerging discourse regarding the risk of deskilling in many professions including physicians from overreliance on AI, particularly among trainees who may forgo independent reasoning (19–22). The current framework, focused on static interactions without modeling temporal skill dynamics, aligns with this concern: high-trust agents showed performance decrements with low-competence AI, suggesting potential overreliance. In training environments, junior clinicians might disproportionately benefit from but also risk dependency on AI assistance. While deskilling is not inevitable, as conditional settings mitigated poor outcomes here, implementation should prioritize strategies like calibrated confidence signals to preserve skills.

### Limitations

While the simulation framework offers valuable insights into human-AI decision-making dynamics, several limitations should be noted. First, the agents in this study are abstracted through fixed parameters for competence, certainty, and trust, which may not fully capture the variability and complexity of real human behavior. In practice, these traits are often context-dependent and influenced by factors such as emotional state, task familiarity, or previous AI interactions. While the current model frames decisions as static binary or ternary choices it abstracts away diagnostic reasoning complexity, such as iterative hypothesis refinement and integration of evolving data. Sequential decision architectures, where prior choices constrain subsequent ones, are prevalent in practice but treated here as decomposable into single events for simulation tractability. Uncertainty is modelled as a fixed trait, though in reality it updates dynamically with new information. These simplifications, typical in initial decision-making simulations, facilitate controlled analysis but may underrepresent authentic clinical environments; future extensions could incorporate more complex architectures. Second, the simulation assumes static AI competence levels and does not account for potential learning or adaptation over time, which limits its applicability to evolving real-world systems. Additionally, the trust parameter is modelled probabilistically without incorporating factors such as explainability, user interface design, or prior AI performance history, which might affect trust in real users. Future work could also include analyses on alternative trust parameterizations, such as asymmetric updating or decay over interactions. The AI confidence signal is also treated as aligned with prediction correctness, which may not reflect actual model calibration or uncertainty estimation challenges. Finally, while the large number of simulated decisions strengthens the reliability of the results, the absence of empirical validation with human subjects means the findings should be interpreted with caution when generalizing to real-world scenarios. Future work should integrate user studies and dynamic trust modelling to further validate and refine these simulation-based insights.

## Conclusion

This study demonstrates that AI assistance can significantly enhance human decision-making, particularly for agents with lower certainty in their own predictions or mismatched competence and confidence. The effectiveness of AI support depends not only on the AI's competence but also on human trust and the complexity of the decision task. Introducing conditional trust improves outcomes by helping human agents calibrate their reliance on AI appropriately. While results show promise, real-world variability in human behavior and system interaction must be addressed through empirical validation and dynamic trust modeling. Ultimately, tailoring AI integration to individual decision-maker profiles and contexts is key to maximizing its value in clinical settings.

## Data Availability

The original contributions presented in the study are included in the article/Supplementary Material, further inquiries can be directed to the corresponding author.

## Supplementary material

Supplementary Figure 1 Decision-making process for a binary trial. Each of the 27 agents is defined by a unique combination of competence (o_a_), certainty (c_a_), and baseline trust (t_a_) at levels 0.3, 0.6, and 0.9. The human first forms a primary decision d_i_(1) (correct with probability o_a_) and may flip it due to uncertainty (probability 1−c_a_). In the no-AI baseline, this post-flip decision d_i_(2) is final. When AI assistance is provided (competence *α*), the model outputs a prediction p_i_ with a correct decision at a probability of *α*. On conflicting decision, the agent adopts the AI's recommendation with probability *τ*_i_ = t_a_.
