# Supplementary material for: AI-supported clinical decision-making: in silico simulation of physician-AI interactions
Source: Front Digit Health. 2026 Jan 9;7:1697825. doi: 10.3389/fdgth.2025.1697825 (PMC12827601; doi:10.3389/fdgth.2025.1697825)
Supplement: Supplementary Figure 1 — Decision-making process for a binary trial. Each of the 27 agents is defined by a unique combination of competence (oa), certainty (ca), and baseline trust (ta) at levels 0.3, 0.6, and 0.9. The human first forms a primary decision di(1) (correct with probability oa) and may flip it due to uncertainty (probability 1−ca). In the no-AI baseline, this post-flip decision di(2) is final. When AI assistance is provided (competence α), the model outputs a prediction pi with a correct decision at a probability of α. On conflicting decision, the agent adopts the AI's recommendation with probability τi = ta. [file Image1.pdf]

Supplementary Figure 1. Decision-making process for a binary trial. Each of the 27 agents is defined by a unique combination of competence (o_a), certainty (c_a), and baseline trust (t_a) at levels 0.3, 0.6, and 0.9. The human first forms a primary decision $d_i^{(1)}$ (correct with probability o_a) and may flip it due to uncertainty (probability $1-c_a$). In the no-AI baseline, this post-flip decision $d_i^{(2)}$ is final. When AI assistance is provided (competence α), the model outputs a prediction p_i with a correct decision at a probability of α. On conflicting decision, the agent adopts the AI's recommendation with probability $\tau_i = t_a$.

True label $y_i \in \{0, 1\}$

Human primary decision $d_i^{(1)} = y_i$ with probability o_a

With probability $1-c_a$ (uncertainty) $\rightarrow d_i^{(2)} \leftarrow 1 - d_i^{(1)}$

AI prediction $p_i = y_i$ with probability $\alpha \in \{0.3, 0.4, 0.5, 0.6, 0.7, 0.8, 0.9\}$

If $p_i \neq d_i^{(2)}$ (conflict):

Adopt p_i with probability $\tau_i = t_a$

Else: Keep $d_i^{(2)}$
